# Narrowband Deep-Blue Multi-Resonance Induced Thermally Activated Delayed Fluorescence: Insights from the Theoretical Molecular Design

**DOI:** 10.3390/molecules27020348

**Published:** 2022-01-06

**Authors:** Yuting Wu, Yanan Zhu, Zewei Zhang, Chongguang Zhao, Junpeng He, Chaoyi Yan, Hong Meng

**Affiliations:** 1 School of Advanced Materials, Peking University Shenzhen Graduate School, Shenzhen 518055, China; wyuting@pku.edu.cn (Y.W.); zhuyn@pku.edu.cn (Y.Z.); 1901213007@pku.edu.cn (Z.Z.); zhaocg15@tsinghua.org.cn (C.Z.); jheorange@pku.edu.cn (J.H.); yancy@pkusz.edu.cn (C.Y.); 2 State High-Tech Industrial Innovation Center, Shenzhen 518057, China

**Keywords:** TADF, multi-resonance, π-conjugate, tunability, molecular design

## Abstract

Multi-resonance thermal activated delayed fluorescence (MR-TADF) has been promising with large oscillator strength and narrow full width at half maxima of luminescence, overcoming the compromise of emission intensity and energy criteria of traditional charge transfer TADF frameworks. However, there are still limited theoretical investigations on the excitation mechanism and systematic molecular manipulation of MR-TADF structures. We systematically study the highly localized excitation (LE) characteristics based on typical blue boron-nitrogen (BN) MR-TADF emitters and prove the potential triangular core with theoretical approaches. A design strategy by extending the planar π-conjugate core structure is proposed to enhance the multiple resonance effects. Moreover, several substituted groups are introduced to the designed core, achieving color-tunable functions with relatively small energy split and strong oscillator strength simultaneously. This work provides a theoretical direction for molecular design strategy and a series of potential candidates for highly efficient BN MR-TADF emitters.

## 1. Introduction

Organic light-emitting diodes (OLEDs) have been drawing attention to the first manufacture of multi-layer thin film structure by Tang and Van Slyke in 1987 [1], inspiring intensive research in optoelectronic applications [2,3,4]. As the key component of OLEDs, the emitter layer plays a critical role in luminescent performance. Since the efficiency of the first-generation OLED is limited by the 25% of singlet excitons [5], phosphorescent OLEDs (PHOLEDs) with metal-complexes performing higher yield (75%) have been proposed. However, the spin-orbit coupling (SOC) effect of PHOLEDs is greatly enhanced due to the effect of heavy atoms, such as iridium and platinum [6]. Nevertheless, PHOLEDs suffer from urgent problems such as the high cost of noble elements, insufficient supplies of rare metals, and non-renewable resources, as well as, most importantly, lack of efficient and stable deep blue emitters [7,8]. Fortunately, thermally activated delayed fluorescence (TADF) has been developed by utilizing inexpensive pure organic materials with great potential in light emission. With a small energy split (∆E_ST_) between S_1_ and T_1_, T_1_ excitons are enabled to convert to S_1_ through reverse intersystem crossing (RISC) under the thermal activation so that TADF-based OLEDs can theoretically achieve 100% internal quantum efficiency (IQE) [9,10,11,12]. Conventional TADF emitters are commonly designed as intermolecular Donor-Acceptor (D-A) systems, in which the highest occupied molecular orbital (HOMO) and the lowest unoccupied molecular orbital (LUMO) are separated by isolating the electron-donating (D) and electron-withdrawing (A) units [13]. However, D-A type TADF molecules have substantial intramolecular charge transfer (ICT) characteristics. The long-distance spatial separation enhances the structural relaxation of excited states, resulting in the expense of low values of oscillator strength (f), larger Stokes-shift, and wider full width at half maxima (FWHM) [14]. In addition, deep blue TADF materials are still a focused-on area. Blue OLED emitters need to satisfy the criteria of wide bandgap generally, between the highest occupied molecular orbital (HOMO) and the lowest unoccupied molecular orbital (LUMO) energy levels, making it challenging to achieve highly efficient and pure blue emission [15].

**Figure 1:**
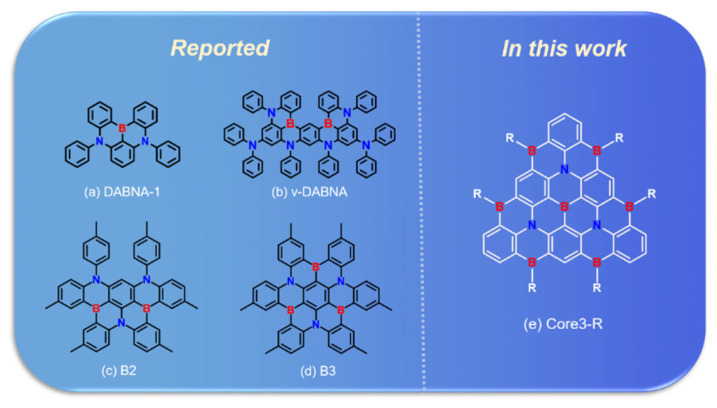
Chemical structures of reported (**a**) DABNA-1, (**b**) ν-DABNA, (**c**) B2, and (**d**) B3 and designed (**e**) Core3-R.

Because of this, in 2016, Hatakeyametal et al., first proposed a new structure of BN-doped multi-resonance (MR) TADF molecule, the DABNA-1 (Figure 1a), with blue emission at 462 nm, and the FWHM of 28 nm [16]. This seminal contribution triggered massive research efforts and opened a new pathway to promote blue light emission with higher color purity. Following this work, in 2019, they reported an emitter ν-DABNA (Figure 1b) with a modified molecular structure and emitting high-purity blue light with an FWHM of 14 nm at 468 nm, which is the narrowest FWHM among all TADF compounds reported so far [17]. In 2018, our group introduced a carbazole unit in the para position of the B-substituted phenyl ring [18]. The resonance effect can be significantly enhanced without compromising the color fidelity, thus improving the performance of the corresponding pure blue TADF-OLED [18]. Furthermore, several strategies for designing MR-TADF molecules have been presented, such as boron-nitrogen (BN) type [18,19,20,21], boron-oxygen type [19,22,23], and boron-carbonyl type [21,24]. Moreover, heterodoped MR-TADF emitters with triangulene backbone of different sizes have been reported [25,26,27,28,29,30]; researchers also synthesized the graphene-like BN-MR-TADF blue molecules B2 and B3 (Figure 1c,d), which are also promising blue TADF materials with small ∆E_ST_ (0.15–0.18 eV) and narrow FWHM (32–34 nm) [31]. The representative MR-TADF compounds are a new class of polycyclic aromatic compounds, designed with fused planar structures containing boron and nitrogen (or oxygen). They have the characteristics of electron donor atoms and electron-deficient atoms disposed para to each other in the polycyclic conjugated hydrocarbons.

In addition, theoretical studies based on DFT level have revealed the different excited process MR-TADF materials, such as the RISC process from T_1_ to S_1_ by Lin and coauthors [32]. Northey et al., identified that the RISC mechanism of DABNA-1 operates through a second-order spin-vibration coupling mechanism like other organic emitters of OLEDs [33]. Furthermore, several new methods have been developed by Pershin et al., which elucidated the local alternating rearrangement of electron density due to MR-TADF excitation using a highly correlated wave function-based method [34]. Recently, Bhattacharyya proposed that DLPNO-STEOM-CCSD computed the more accurate singlet-triplet energy splitting for the studied system [35]. However, to simulate the spectra and excitation energy, some functionals of DFT level can give a reasonable prediction. For example, the calculation of DABNA-1 with O3lyp has been confirmed by the reported results [32].

**Figure 2:**
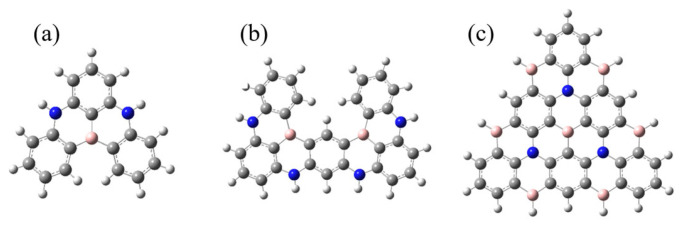
The structure of CoreN: (**a**) Core1, (**b**) Core2, and (**c**) Core3.

In this work, based on classical blue BN-MR-TADF emitters DABNA-1 and ν-DABNA, we extracted their resonant cores and named them Core1 and Core2, depicted in Figure 2a,b, respectively. To design a high-efficiency deep blue light emission theoretically, the fused multi-resonant core should be extended to achieve a shorter-range electron reorganization. We design a π-conjugate triangular graphene-like BN-MR-TADF system called Core3-R in Figure 1e. It exhibits higher localized excitation (LE) characteristics compared to the classical BN-MR-TADF molecules. The extension of the MR-effect conjugate resonance structure enables less energy relaxation loss in the non-radiative transition process, ensuring the effective transmission between electron and hole to improve the radiative energy utilization of the emitting molecules. Moreover, we further design color-tunable molecules by introducing different substituents based on the Core3 system, simultaneously attaining small energy split and strong oscillator strength.

## 2. Computational Methods

Quantum chemical calculations are performed with Gaussian 16 program package [36]. In this work, vibrational frequencies are calculated for all optimized structures, and their analyses show no imaginary frequencies. Ground state geometric optimization calculations are based on the density functional theory (DFT) method, with the hybrid functional B3LYP and the 6–31G(d) basis set [37]. Accounting for van der Waals and other dispersion weak interaction forces, the DFT-D3 empirical dispersion correction method with the Becke-Johnson damping model is also included in the calculations [38,39].

The geometry optimizations for excited states and the calculations of vertical transition energy are carried out with the O3LYP hybrid functional at the basis set level of 6-311G(d,p), because their calculated results are in better agreement with the experimental data in DABNA-1, v-DABNA, B2, and B3, as verified in Table 1. The absorption values extracted from vertical excitation are determined utilizing time-dependent density functional theory (TD-DFT) calculation (Figure S1 and Tables S1–S3 in the Supporting Materials), and the geometry of the S_1_ state is also optimized to obtain the emission values without solvent (Table S2). Wave function analysis, including hole-electron localization [40], is processed by the multi-functional wave function analyzer code, Multiwfn (Version 3.8) [41]. The results are plotted via Visual Molecular Dynamic (VMD) software [42].

Moreover, the calculations of the CoreN (N = 1,2,3) and Core3-R (Core3-2F: R = ortho-fluorophenyl, Core3-4F: R = para-fluorophenyl, Core-CF3: R = trifluoromethyl, Core3-CZ: R = carbazole) molecules are also carried out with O3LYP/6-311G (d). The detailed design structure of Core3-R is in Figure S5. The introduction of strong electron-withdrawing group (trifluoromethyl) and strong electron-donating group (carbazole), small steric effect (trifluoromethyl) and large steric effect (carbazole), and different substitution sites (ortho or para fluorophenyl), etc., not only explores the effect of discrepant electronic effects and steric effect on the system but can also contribute to extend the color tunability application.

## 3. Results and Discussion

### 3.1. Structure and Spectra

**Figure 3:**
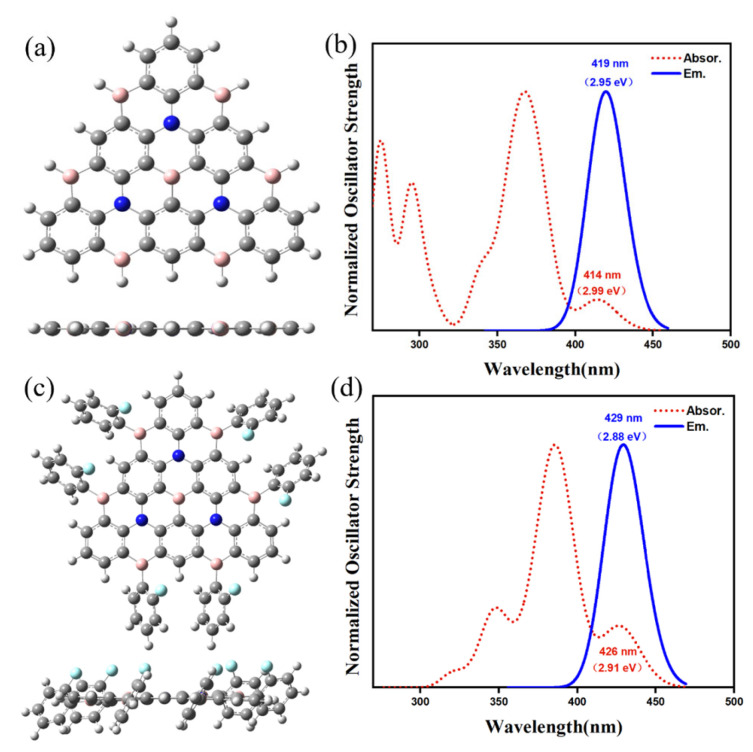
The structure (**a**) and spectra (**b**) of Core3, and the structure (**c**) and spectra (**d**) of Core3-2F.

The ground state structures of Core3 (R = H) and Core3-2F (R = ortho fluorophenyl) are shown in Figure 3a,c, respectively. It shows that the geometry of Core3 looks like an equilateral triangle with a slight twist angle of about 0.02°, maintaining a planar conjugated structure of B, N atoms, and benzene rings interleaving. Compared with Core3, the conjugate core configuration of Core3-2F with fluorophenyl substituents have an insignificant variation of dihedral angles, maintaining the almost planar structure. As the theoretical spectra shown in Figure 3b,d, the absorption and emission wavelengths of Core3 are 414 nm and 419 nm, and that of Core3-2F is 426 nm and 429 nm, indicating that the two molecules designed here are potentially deep blue light emitters with a slight Stokes shift (≤5 nm). Moreover, it is of interest to observe that the HOMO and LUMO of Core3-2F (Figure S3 in Supporting Materials). The electron density is almost entirely distributed on the conjugated center, Core3, without different extensions to the substituents, which is also consistent with their very similar calculated spectra. Analogously, we find that the electron density of classical MR-BN-TADF molecules DABNA-1 and ν-DABNA are localized on the conjugate plane of the non-substituted group (Figure S4 in Supporting Materials). Therefore, the investigation for the properties of the excited states of BN-MR-TADF systems is based on those typical Cores.

As expected, Core3 has a large number of degenerate states in the orbitals of different excited states because of the high symmetry of the system. For example, the excitation energy, absorption wavelength, and oscillator strength of S1 and S2, S4, and S5 states are almost the same values (Table S2 in the Supporting Materials). It reveals that the structure of Core3 tends to be more planar than Core1 and Core2, for which the lone pair electrons of the nitrogen atoms in the triangle plane can better participate in the π-system. Moreover, due to the conjugated rigid molecular skeleton, the structural relaxation is dramatically suppressed. We can conclude that the extension of π-conjugation is a valuable method to reduce the off-diagonal vibration coupling constant (VCCS) [38]. By introducing N or B into the conjugated plane, the constructive π-conjugated planar structure could benefit the multi-resonance effect and the deep blue emission.

**Figure 4:**
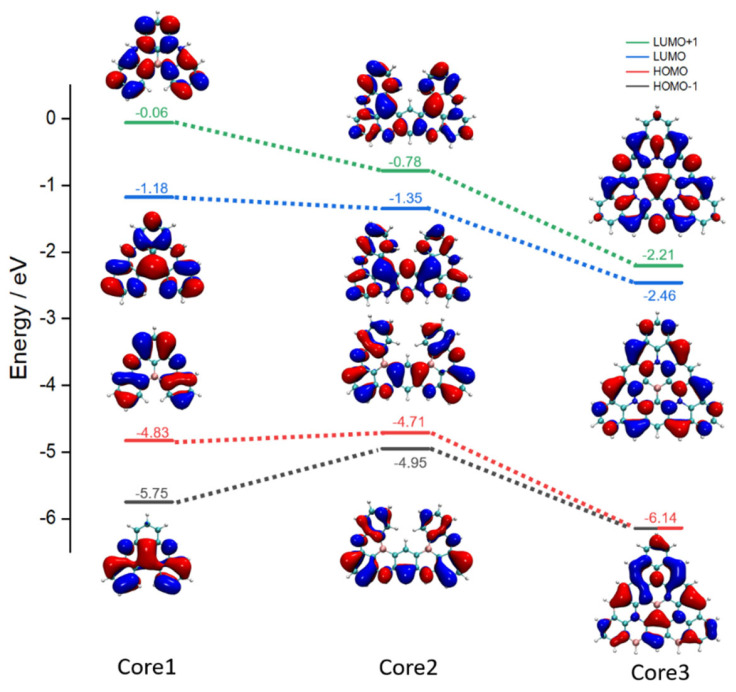
Calculated energy levels and electron distribution of the HOMO-1, HOMO, LUMO, LUMO+1 for Core1, Core2, and Core3 in the ground state, respectively. The iso-surface value is 0.02 eV.

To explore the energy arrangement and the multi-resonance regularity in the BN-MR-TADF system, the energy level and distribution characteristics for the frontier molecular orbital HOMO-1, HOMO, LUMO, and LUMO+1 of Core1, Core2, and Core3 are displayed in Figure 4. As for the energy level, we can see that the overall orbital energy level of the Core3 system is significantly lower than that of Core1 and Core2. Nevertheless, the energy gap of Core3 increases to 3.68 eV, indicating that Core3 might be a deep blue emitter. With the more extended resonance system configuration from Core1 to Core3, the levels between LUMO and LUMO+1, HOMO, and HOMO-1 become closer, like the HOMO and HOMO-1 become degenerate with an energy level of −6.14 eV. Furthermore, the Core3 system maintains the multi-resonance cooperative pattern of atom-local delocalization. Compared with Core1 and Core2, the electron density of Core3 exhibits a more short-range separation and uniform electronic distribution, thus reducing the energy dissipation caused by non-radiative transition during the excitation process, ensuring a high oscillator strength of 0.1323 for Core3 during the first transition process from S_1_ to S_0_. (Oscillator strength can be found in Table S4 of Supporting Materials).

### 3.2. Hole-Electron Analysis

The realization of the unique narrow-band deep blue spectrum of BN-TADF requires further analysis of the excited state. It is well known that the electron excitation processes can be described as the hole-to-electron transition [40,43]. To further investigate the characteristics of electron excitation in-depth, we analyze the hole-electron distribution quantitatively during the singlet excitation, and the output information is illustrated in Table 2. There are several indices such as D-index (the distance between the hole and the electron centroid), Sr-index (the overlapping degree of hole and electron), T-index (the degree of separation between hole and electron), HDI (the hole delocalization index), EDI (the electron delocalization index), and orbital contributions.

As given in Table 2, the D-index of Core3 shows a small value of 0.356 Å, less than a third of the length of the carbon-carbon bond in benzene (1.39 Å), which indicates that the distance of the centroids between the hole and electron is close to extreme. The D-index values of Core1 and Core2 are 1.71 Å and 0.558 Å, respectively, which are larger than that of Core3, denoting shorter transmission distance along with less energy loss in Core3 and implying its higher luminous efficiency. In addition, the normalized Sr-index value of Core3 reaches 0.78 a. u., indicating that more than three-quarters of the distribution characteristics of hole and electron overlap perfectly, which is larger than the Sr-index of Core1 (0.63 a. u.) and Core2 (0.62 a. u.). From the Sr-index and D-index, it can be judged that Core3 has a highly localized excitation (LE) characteristic. Next, the T-index of Core3 is −2.81 Å, which is significantly more negative than Core1 (−0.31 Å) and Core2 (−1.63 Å). It means that in Core3 there is no apparent separation between the overall distribution of holes and electrons, which also satisfies the excitation characteristics of LE, and indicates a higher degree of hole and electron delocalization in the Core3. These index values results provide a quantitative analysis of the hole and electron distribution and demonstrate a higher short-range separation and more uniform electronic distribution in the Core3 system to achieve a more efficient deep blue emission.

We also note that the orbital contributions to hole and electron of Core1 and Core2 almost entirely come from HOMO and LUMO orbitals. However, for the designed Core3 system, despite the maximum contribution for the hole of 76.51% coming from HOMO orbital (MO145), 22.20% of hole density is composed of HOMO-1 orbital. At the same time, the electron density contribution of Core3 is almost the same as that of Core1 and Core2. In a more extended conjugation system provided by Core3, the BN cooperative resonance concurrently reduces the LUMO+1 energy level and increases the HOMO-1 energy level. As a result, HOMO-1 and LUMO+1 orbitals are closer to the LUMO and HOMO orbital energy levels, respectively, and contribute to the spectrum. Moreover, the analysis of MO145 (HOMO-1), 146 (HOMO), 147 (LUMO), and 148 (LUMO+1) can provide a qualitative description of the electron excitation of the Core3 system. It can be seen from Figure S3 that the distribution is all local atomic distribution, consistent with the higher excitation characteristics of LE.

**Figure 5:**
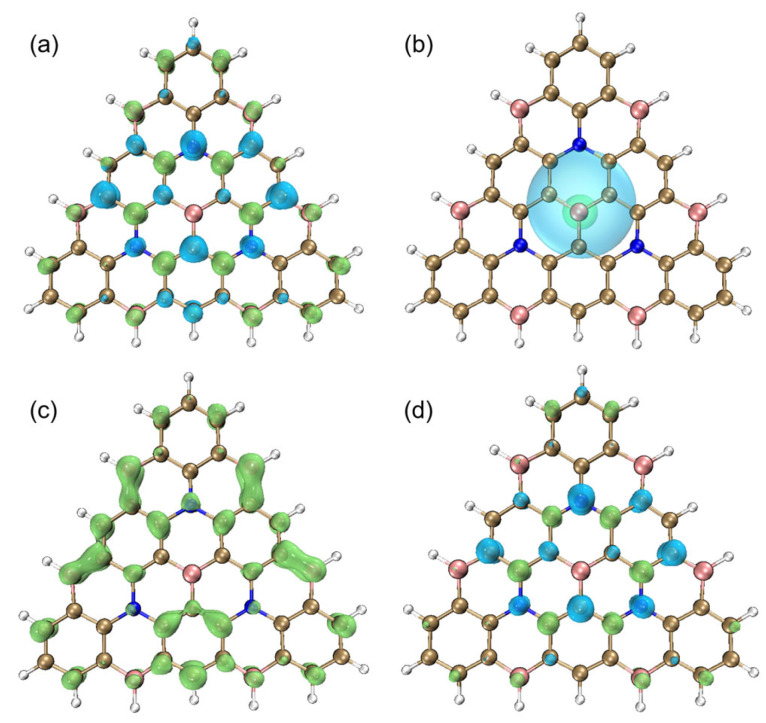
(**a**) The hole and electron diagram, (**b**) C_hole_ and C_ele_ diagram, and (**c**) Sr(R) diagram of Core3, where electron distribution is shown in green, and hole distribution is shown in blue. (**d**) The density difference (CDD) diagram of Core3, where green and blue correspond to the increase and decrease of the first excited state density relative to the ground state density, respectively. The iso-surface value is 0.002 eV.

Figure 5 shows the images of the hole and electron distribution of Core3, where electron distribution is shown in green, and hole distribution is shown in blue. Figure 5a shows that the hole and electron distribution generally present local interval distribution one by one. It obtains intuitively the localized excitation feature of the Core3 system, which verifies our inferences based on D, Sr, and T indices. At the same time, the more short-range transmission charge can reduce the energy loss caused by non-radiative transition due to structural relaxation, better realizing the deep blue emission. The C_hole_ and C_ele_ diagram describes the overall trend distribution of holes and electrons by Gaussian functions, erasing the distribution details. This makes the equivalent surface maps of holes and electrons into a continuous shape, more straightforward, and more intuitive. As shown in Figure 5b, the contour surface of the hole and electron seems to be close to the circle, which signifies the almost isotropic distribution of hole and electron, which is consistent with the symmetric nature of the atomic distribution. The distribution of holes is broader than that of electrons. Next, Sr(R) is the overlap function diagram of holes and electrons. From Figure 5c, we can see that hole and electron overlap at most atoms. Interestingly, the overlapping scope is limited to a single atom, thus achieving a more localized and individual separation between holes and electrons. Lastly, the charge density difference (CDD) diagram between the first excited state and the ground state is shown in Figure 5d. Green and blue correspond to the increase and decrease of the excited state density relative to the ground state density, respectively. Notably, the electron regions are mainly located on the nitrogen atoms, and the hole regions are localized on the boron atoms. The Core3 maintains the unique density distribution and multiple synergistic resonance excitations of the MR-BN-TADF system. Core3 achieves a shorter-range density recombination transition of higher LE characteristics with highly efficient blue light excitation due to the extended plane conjugate structure.

**Figure 6:**
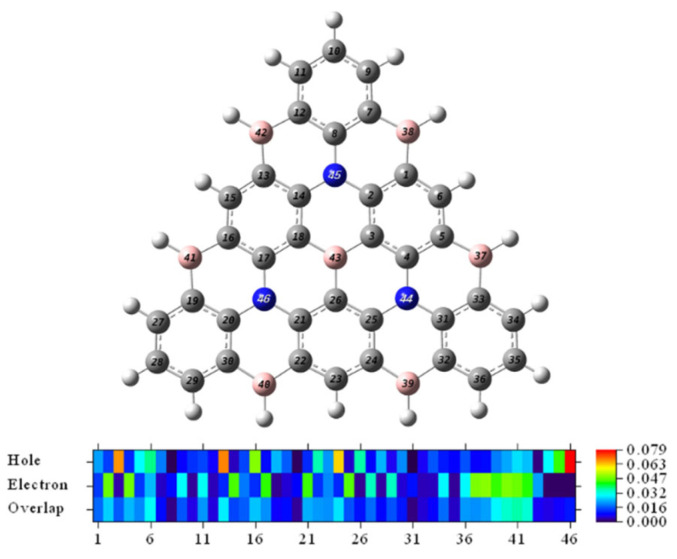
Heat map representation of the hole-electron distribution.

To more accurately quantify the contributions of individual atoms (except hydrogen atoms) to holes and electrons, we calculated the contributions of individual atoms (Table S3 in the Supporting Materials) and plotted the heat map by numerical size (Figure 6). It can be seen from the color that the delocalization characteristics of the electron distribution are more robust than the holes. The total value of the top 5 atoms with the highest contribution ratio of holes was 32.89%, and the total contribution value of electrons was 24.61%. So, the hole was not as concentrated as the electrons. The sum of the difference value of the 13 atoms in the core of center B and 3N atoms is −2.82%, and the integral value of the density difference is negative. It is evidenced that the resonance core of the center is electron-free, and the electrons tend to be transferred to the peripheral atoms in the excitation process. The hole characteristic of the center is obvious evidence, which is following the result of the C_hole_ and C_ele_ diagram.

### 3.3. Color Tunability over the Spectrum

**Figure 7:**
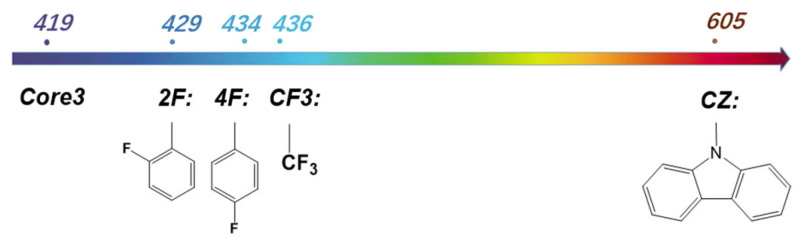
The molecular emission wavelength was introduced to the Core3 system by different substituents.

Herein, Core3 based on our design shows the unique excitation property of realizable deep blue light with a small Stokes-shift. But except for deep blue light, the different emission wavelengths can be achieved by the further regulation of molecular structure, for example, introducing an electron-donating or electron-withdrawing group outside the conjugate plane. As presented in Figure 7, blue emitters can be designed by introducing strong electron-withdrawing groups such as trifluoromethyl, ortho, and para fluorophenyl, with respective light-emission at the wavelength of 436 nm, 434 nm, and 429 nm, and immense emission oscillator strength of 0.0741, 0.1323, and 0.1449. As the substitution changes, the planarity of the core structure adjusts accordingly. With the torsion angle of the core conjugate plane increasing, the conjugate MR effect weakens, resulting in a smaller gap and redshift of the spectrum (Figure S6 in Supporting Materials). On the other hand, red-emissive candidates are designed by introducing strong electron-donating groups. For example, Core3-CZ, comprising Core3 and a carbazole unit, emits at the wavelength of 605 nm with an oscillator strength of 0.0877. Therefore, utilizing Core3 to build an emissive molecule system is a promising strategy to obtain feasible color-tunable full spectrum regulation by adjusting the substituent groups with discrepant electronic effects.

The theoretical values of ∆E_ST_ for studied molecules in Table 3 infer that the molecule is potential TADF candidate. The smaller the energy split between S_1_ and T_1_ states is, the more favorable it is for excitons to realize the RISC crossover from the triplet to singlet states and realize a more efficient TADF emission. Generally, the adiabatic ∆E_ST_ value conforms more to the actual excitation process from the theoretical perspective. The adiabatic energy values here are obtained from the optimized S_1_ and T_1_ states, and details are found in Supporting Materials. As depicted in Table 3, the respective experimental ∆E_ST_ of typical BN-MR-TADF molecules DABNA-1 and ν-DABNA are 0.18 eV [16] and 0.017 eV [17], and their simulated adiabatic ∆E_ST_ values are 0.31 eV and 0.23 eV, respectively. This result is consistent with the reported literature [17], where the reported theoretical ΔE_ST_ values are generally larger than the experimental values, regardless of the choice of DFT function and the use of the Tamm-Dancoff approximation. The theoretical ∆E_ST_ values of designed molecules Core3, Core3-CZ, Core3-2F, Core3-4F, and Core3-CF3 are 0.24 eV, 0.32 eV, 0.21 eV, 0.21 eV, and 0.23 eV, respectively. These results are close to that of the typical BN-MR-TADF molecular values at the same computational level. It is speculated that the derivatives of Core3 are capable of being TADF candidates, with small enough ∆E_ST_ and strong oscillator strength simultaneously.

## 4. Conclusions

In conclusion, we systematically studied the highly localized excitation characteristics of typical MR-BN-TADF molecules and proposed promising candidates with blue emission with DFT approaches and electron-hole analysis. The recombination of electrons and holes in the shorter range can reduce the energy loss caused by structural relaxation during the excitation process and achieve emission with more efficiency. By extending the planar conjugation size and alternating electron-donor/acceptor substituents based on the MR-core, a design strategy to enhance the LE characteristics of TADF molecules has been presented. Simultaneously, strong oscillator intensity and small ∆E_ST_ can be exhibited in the BN-MR-TADF molecules designed here. Moreover, the substituent groups introduced here are supposed to realize color tunability across the spectrum. Overall, this work can guide developing potential TADF candidates with high efficiency and color purity for the next generation.

## Figures and Tables

**Table 1:** Comparison between the experimental and calculated wavelength of DABNA-1, v-DABNA, B2, and B3, in the computational level of O3LYP/6-311G (d) without solvent.

Compound	Experimental		Calculated	
	λ_[Abs]_ (nm)	λ_[PL]_ (nm)	λ_[Abs]_ (nm)	λ_[Em]_ (nm)
DABNA-1	437 [16]	462 [16]	420	459
v-DABNA	457 [17]	468 [17]	458	468
B2	438 [31]	462 [31]	438	455
B3	396 [31]	455 [31]	396	441

**Table 2:** The excited-state analysis is based on values for the D, Sr, H, t, HDI, EDI, and orbital contribution for the dominant transitions in Core1, Core2, and Core3, respectively.

Core	D (Å)	Sr	H (Å)	T (Å)	HDI	EDI	Orbital Contribution	
							Hole	Electron
Core1	1.71	0.63	3.20	−0.31	7.74	5.81	MO70: 97.76%	MO71: 97.21%
Core2	0.558	0.62	4.56	−1.63	5.55	4.79	MO119: 97.98%	MO120: 97.89%
Core3	0.356	0.78	4.55	−2.81	4.43	3.78	MO145: 76.51%	MO146: 76.93%
							MO144: 22.19%	MO147: 21.87%

**Table 3:** ∆E_ST_ comparison for reported DABNA-1, ν-DABNA, and molecules we designed, in the computational level of O3LYP/6-311G (d) without solvent.

Compound	∆E_ST [CALC]_ (eV)		∆E_ST [EXP]_ (eV)
	Vertical Energy	Adiabatic Energy	
DABNA-1	0.43	0.31	0.18 [16]
ν-DABNA	0.27	0.23	0.017 [17]
Core3	0.29	0.24	-
Core3-2F	0.27	0.21	-
Core3-4F	0.22	0.21	-
Core3-CF3	0.29	0.23	-
Core3-CZ	0.29	0.32	-

## Data Availability

Data is contained within the article or Supplementary Material.

## Supplementary Materials

The following are available online, Figure S1: The absorption spectra; Figure S2: Ground state structure and energy level of Core1, Core2 and Core3; Figure S3: The main contributing orbitals of hole and electron of Core3-2f; Figure S4: The distribution of HOMO (a), LUMO (b) of DABNA-1, and distribution of HOMO (c), LUMO (d) of v-DABNA; Figure S5. The designed structure of Core3-2F (a), Core3-4F (b), Core3-CF3 (c) and Core3-CZ (d); Figure S6: The structure and twist angle of Core3-2F (a), Core3-4F (b), Core3-CF3 (c) and Core3-CZ (d); Table S1: Comparison between experimental and calculated wavelength of DABNA-1 and v-DABNA; Table S2: The excited energy (eV), wavelength (nm), frequency (1000 cm^−1^) and oscillator strength of different excited states for Core3; Table S3: The percentage of holes or electrons each atom contributes of Core3; Table S4: Emission values of Core1, Core2 and Core3.

## Sample Availability

Samples of the compounds are not available from the authors.
