# Supplementary material for: Narrowband Deep-Blue Multi-Resonance Induced Thermally Activated Delayed Fluorescence: Insights from the Theoretical Molecular Design
Source: Molecules. 2022 Jan 6;27(2):348. doi: 10.3390/molecules27020348 (PMC8780697; doi:10.3390/molecules27020348)
Supplement: Supplementary file 1 [file molecules-27-00348-s001.zip › molecules-1533003-supplementary.pdf]

Supporting Information

Narrowband Deep-blue Multi-Resonance Induced Thermally Activated Delayed Fluorescence: Insights from the Theoretical Molecular Design

Yuting Wu [a], Yanan Zhu [a], Zewei Zhang [a], Chongguang Zhao [a,b], Junpeng He[a], Chaoyi Yan[a], and Hong Meng *[a]

a. School of Advanced Materials, Peking University Shenzhen Graduated School, Peking University, Shenzhen 518055, China. Email: *menghong@pku.edu.cn*

b. State High-Tech Industrial Innovation Center, Shenzhen 518057, China.

1. Absorption Values

(Base on optimized ground structure)

Key words: #p 6-311g(d) o3lyp TD(nstates=50) IOp(9/40=4)

multiplicity = 1

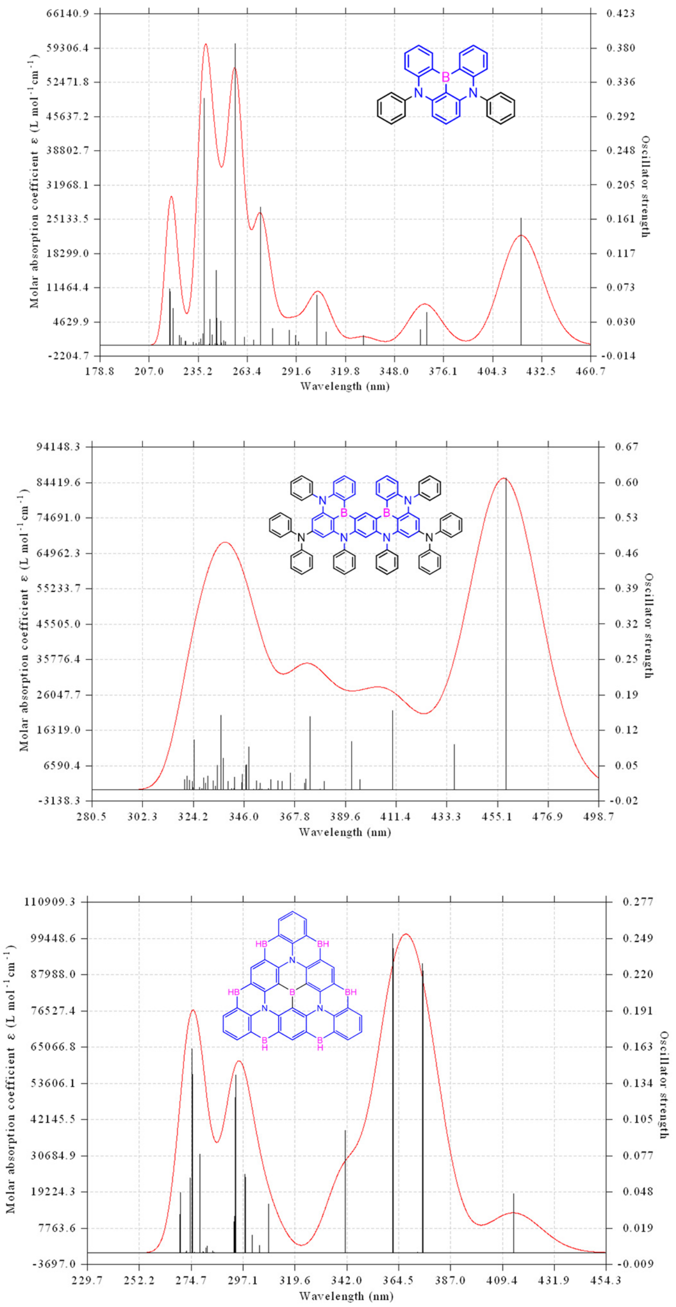

Figure S1. The absorption spectra.

Table S1. Comparison between experimental and calculated wavelength of DABNA-1 and v-DABNA, in the computational level of B3LYP/6-31G (d).

Compound	Experimental		Calculated	
	$\lambda_{[Abs]}$ (nm)	$\lambda_{[PL]}$ (nm)	$\lambda_{[Abs]}$ (nm)	$\lambda_{[Em]}$ (nm)
DABNA-1	437 [16]	462 [16]	396	428
v-DABNA	457 [17]	468 [17]	427	437

Table S2. The excited energy (eV), wavelength (nm), frequency (1000 cm-1) and oscillator strength of different excited states for Core3. (Calculated by Multiwfn)

Index	Excit.energy	nm	1000 cm^-1	Oscil.str.	Index	Excit.energy	nm	1000 cm^-1	Oscil.str.
1	2.99170	414.42725	24.12969	0.04770	26	4.16470	297.70260	33.59057	0.06390
2	2.99170	414.42725	24.12969	0.04770	27	4.19550	295.51710	33.83899	0.00000
3	3.22810	384.07794	26.03638	0.00000	28	4.22180	293.67616	34.05112	0.00000
4	3.31110	374.45018	26.70583	0.23880	29	4.22190	293.66920	34.05192	0.00000
5	3.31110	374.45018	26.70583	0.23880	30	4.22280	293.60661	34.05918	0.16130
6	3.31190	374.35973	26.71228	0.00000	31	4.22290	293.59966	34.05999	0.16130
7	3.33450	371.82246	26.89456	0.00000	32	4.32960	286.36410	34.92058	0.00000
8	3.43270	361.18566	27.68660	0.23710	33	4.33720	285.86231	34.98188	0.00000
9	3.43270	361.18566	27.68660	0.23720	34	4.36520	284.02868	35.20772	0.00000
10	3.54000	350.23785	28.55203	0.00000	35	4.36520	284.02868	35.20772	0.00000
11	3.64010	340.60658	29.35939	0.09700	36	4.41670	280.71682	35.62309	0.00550
12	3.64010	340.60658	29.35939	0.09700	37	4.41670	280.71682	35.62309	0.00550
13	3.85340	321.75274	31.07977	0.00000	38	4.41790	280.64058	35.63277	0.00000
14	3.87620	319.86017	31.26366	0.00000	39	4.41790	280.64058	35.63277	0.00000
15	3.87620	319.86017	31.26366	0.00000	40	4.45500	278.30348	35.93200	0.09220
16	3.91880	316.38308	31.60726	0.00000	41	4.45500	278.30348	35.93200	0.09220
17	4.01520	308.78711	32.38478	0.00000	42	4.50130	275.44087	36.30544	0.00000
18	4.02280	308.20374	32.44607	0.03740	43	4.51110	274.84250	36.38448	0.00000
19	4.02290	308.19608	32.44688	0.03740	44	4.51530	274.58685	36.41835	0.18860
20	4.07790	304.03933	32.89048	0.00510	45	4.51530	274.58685	36.41835	0.18860
21	4.07790	304.03933	32.89048	0.00510	46	4.52210	274.17395	36.47320	0.00000
22	4.11680	301.16644	33.20423	0.01120	47	4.54650	272.70252	36.67000	0.00000
23	4.11690	301.15912	33.20504	0.01120	48	4.54660	272.69652	36.67081	0.00000
24	4.12750	300.38571	33.29054	0.00000	49	4.59410	269.87702	37.05392	0.04360
25	4.16470	297.70260	33.59057	0.06390	50	4.59420	269.87114	37.05473	0.04350

Table S3. The percentage of holes or electrons each atom contributes of Core3. The sum of hole and electron showed above is both 100.00%. (Calculated by Multiwfn)

```
 1(C ) Hole: 2.24 % Electron: 1.39 % Overlap: 1.76 % Diff.: -0.85 %
 2(C ) Hole: 1.40 % Electron: 4.81 % Overlap: 2.60 % Diff.:  3.41 %
 3(C ) Hole: 6.79 % Electron: 0.39 % Overlap: 1.62 % Diff.: -6.40 %
 4(C ) Hole: 1.19 % Electron: 4.85 % Overlap: 2.41 % Diff.:  3.66 %
 5(C ) Hole: 2.96 % Electron: 1.37 % Overlap: 2.01 % Diff.: -1.59 %
 6(C ) Hole: 3.76 % Electron: 2.46 % Overlap: 3.04 % Diff.: -1.31 %
 7(C ) Hole: 1.91 % Electron: 0.51 % Overlap: 0.98 % Diff.: -1.40 %
 8(C ) Hole: 0.06 % Electron: 0.99 % Overlap: 0.25 % Diff.:  0.93 %
 9(C ) Hole: 0.86 % Electron: 3.01 % Overlap: 1.61 % Diff.:  2.15 %
10(C ) Hole: 1.30 % Electron: 0.32 % Overlap: 0.65 % Diff.: -0.98 %
11(C ) Hole: 1.49 % Electron: 3.06 % Overlap: 2.13 % Diff.:  1.57 %
12(C ) Hole: 0.80 % Electron: 0.46 % Overlap: 0.61 % Diff.: -0.34 %
13(C ) Hole: 6.88 % Electron: 1.19 % Overlap: 2.86 % Diff.: -5.69 %
14(C ) Hole: 0.49 % Electron: 4.57 % Overlap: 1.49 % Diff.:  4.08 %
15(C ) Hole: 1.48 % Electron: 1.89 % Overlap: 1.68 % Diff.:  0.41 %
16(C ) Hole: 5.02 % Electron: 1.27 % Overlap: 2.52 % Diff.: -3.75 %
17(C ) Hole: 0.98 % Electron: 4.44 % Overlap: 2.08 % Diff.:  3.46 %
18(C ) Hole: 2.40 % Electron: 0.28 % Overlap: 0.83 % Diff.: -2.12 %
19(C ) Hole: 1.66 % Electron: 0.57 % Overlap: 0.97 % Diff.: -1.09 %
20(C ) Hole: 0.11 % Electron: 0.93 % Overlap: 0.32 % Diff.:  0.82 %
21(C ) Hole: 1.22 % Electron: 4.49 % Overlap: 2.34 % Diff.:  3.26 %
22(C ) Hole: 3.58 % Electron: 1.30 % Overlap: 2.16 % Diff.: -2.27 %
23(C ) Hole: 2.12 % Electron: 2.02 % Overlap: 2.07 % Diff.: -0.10 %
24(C ) Hole: 6.29 % Electron: 1.21 % Overlap: 2.76 % Diff.: -5.08 %
25(C ) Hole: 0.54 % Electron: 4.66 % Overlap: 1.58 % Diff.:  4.12 %
26(C ) Hole: 3.64 % Electron: 0.31 % Overlap: 1.06 % Diff.: -3.33 %
27(C ) Hole: 0.91 % Electron: 3.47 % Overlap: 1.77 % Diff.:  2.57 %
28(C ) Hole: 2.38 % Electron: 0.37 % Overlap: 0.93 % Diff.: -2.02 %
29(C ) Hole: 0.71 % Electron: 3.45 % Overlap: 1.56 % Diff.:  2.74 %
30(C ) Hole: 2.04 % Electron: 0.58 % Overlap: 1.09 % Diff.: -1.46 %
31(C ) Hole: 0.05 % Electron: 1.03 % Overlap: 0.22 % Diff.:  0.98 %
32(C ) Hole: 0.74 % Electron: 0.44 % Overlap: 0.57 % Diff.: -0.30 %
33(C ) Hole: 1.59 % Electron: 0.48 % Overlap: 0.87 % Diff.: -1.11 %
34(C ) Hole: 1.10 % Electron: 2.90 % Overlap: 1.78 % Diff.:  1.81 %
35(C ) Hole: 0.88 % Electron: 0.32 % Overlap: 0.53 % Diff.: -0.56 %
36(C ) Hole: 1.58 % Electron: 2.93 % Overlap: 2.15 % Diff.:  1.35 %
37(B ) Hole: 0.86 % Electron: 4.96 % Overlap: 2.06 % Diff.:  4.10 %
38(B ) Hole: 0.98 % Electron: 5.07 % Overlap: 2.23 % Diff.:  4.09 %
39(B ) Hole: 1.92 % Electron: 4.45 % Overlap: 2.92 % Diff.:  2.53 %
40(B ) Hole: 2.30 % Electron: 4.96 % Overlap: 3.38 % Diff.:  2.66 %
41(B ) Hole: 2.73 % Electron: 4.81 % Overlap: 3.63 % Diff.:  2.08 %
42(B ) Hole: 2.48 % Electron: 4.41 % Overlap: 3.31 % Diff.:  1.93 %
43(B ) Hole: 0.24 % Electron: 2.22 % Overlap: 0.73 % Diff.:  1.98 %
44(N ) Hole: 2.98 % Electron: 0.13 % Overlap: 0.63 % Diff.: -2.85 %
45(N ) Hole: 4.47 % Electron: 0.14 % Overlap: 0.78 % Diff.: -4.33 %
46(N ) Hole: 7.91 % Electron: 0.15 % Overlap: 1.08 % Diff.: -7.76 %
```

2. Emission Values

#p opt TD o3lyp/6-311g(d)

multiplicity = 1

Table S4. Emission values of Core1, Core2 and Core3.

Cores	Excit.energy(eV	nm	Oscil.str.
1	2.6927	460.45	0.0808
2	2.6471	468.37	0.1265
3	2.9544	419.66	0.1323

3. Ground states

Key words: #p opt freq b3lyp/6-31g(d) EmpiricalDispersion=GD3BJ

multiplicity = 1

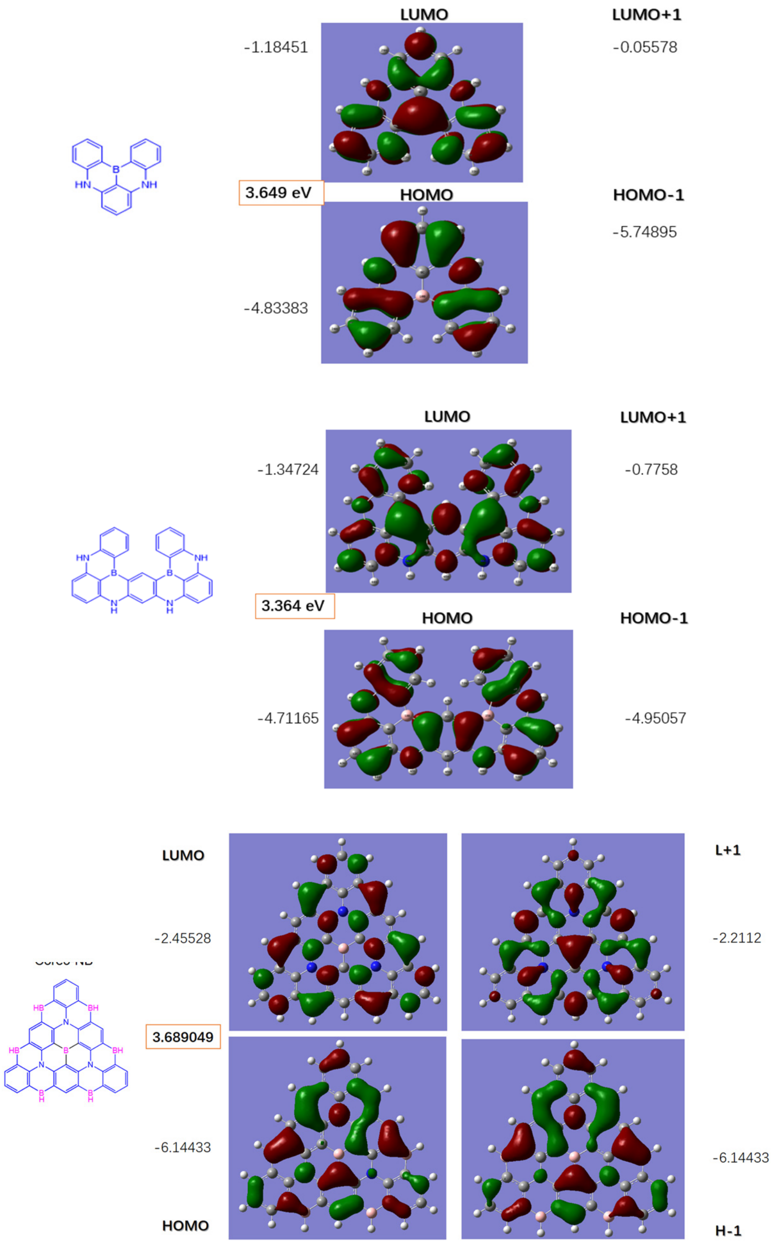

Figure S2. Ground state structure and energy level of Core1, Core2 and Core3.

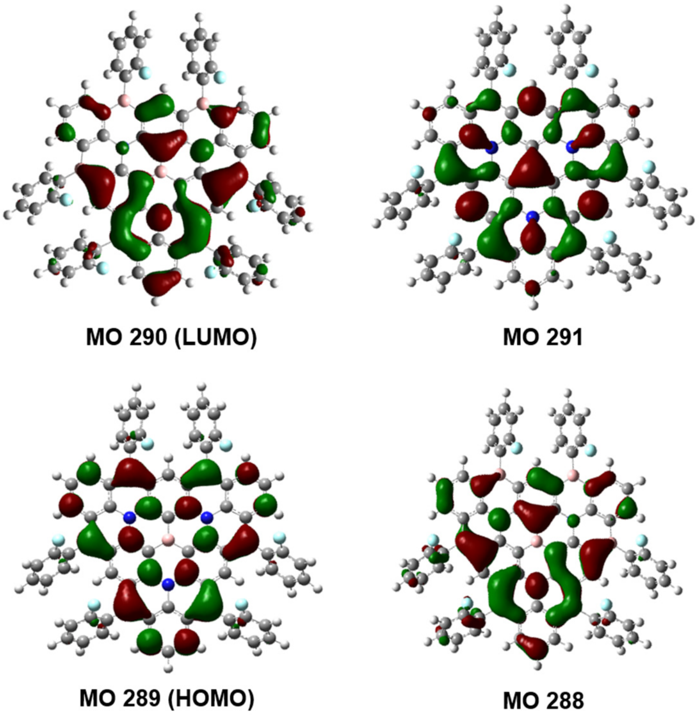

Figure S3. The main contributing orbitals of hole and electron of Core3-2f. (MO 288, Hole: 14.618 %, Electron: 0.000 %; MO 289, Hole: 83.916 %, Electron: 0.000 %; MO 290, Hole: 0.000 %, Electron: 83.963 %; MO 291, Hole: 0.000 %, Electron: 14.804 %.)

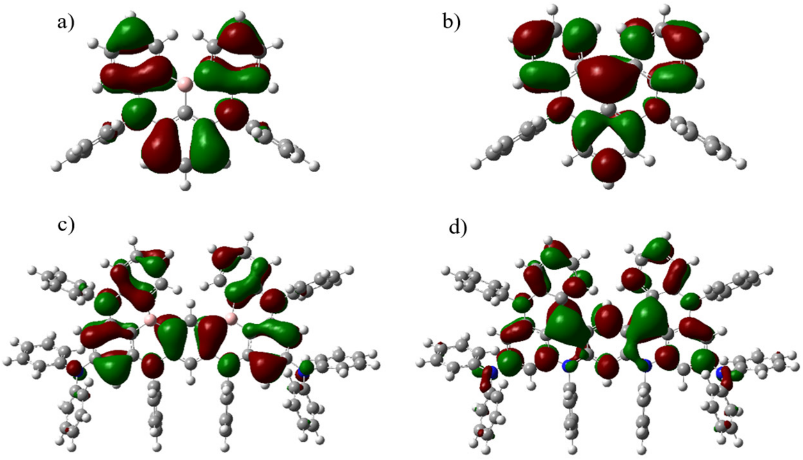

Figure S4. The distribution of HOMO (a), LUMO (b) of DABNA-1, and distribution of HOMO (c), LUMO (d) of v-DABNA. The results are performed in B3LYP-D3(BJ)/6-31G(d) with Gaussian 16 package.

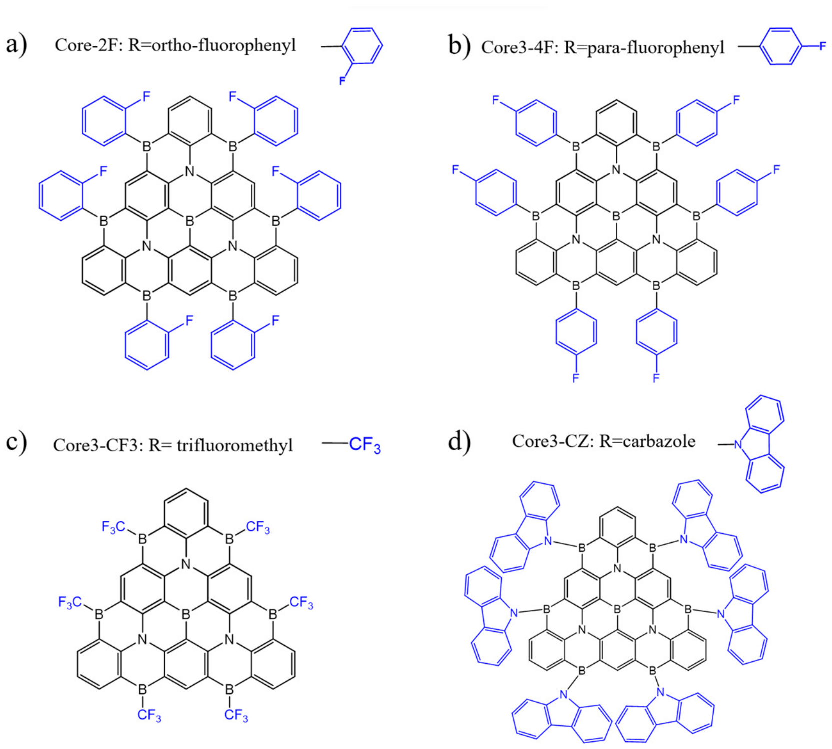

Figure S5. The designed structure of Core3-2F (a), Core3-4F (b), Core3-CF3 (c) and Core3-CZ (d).

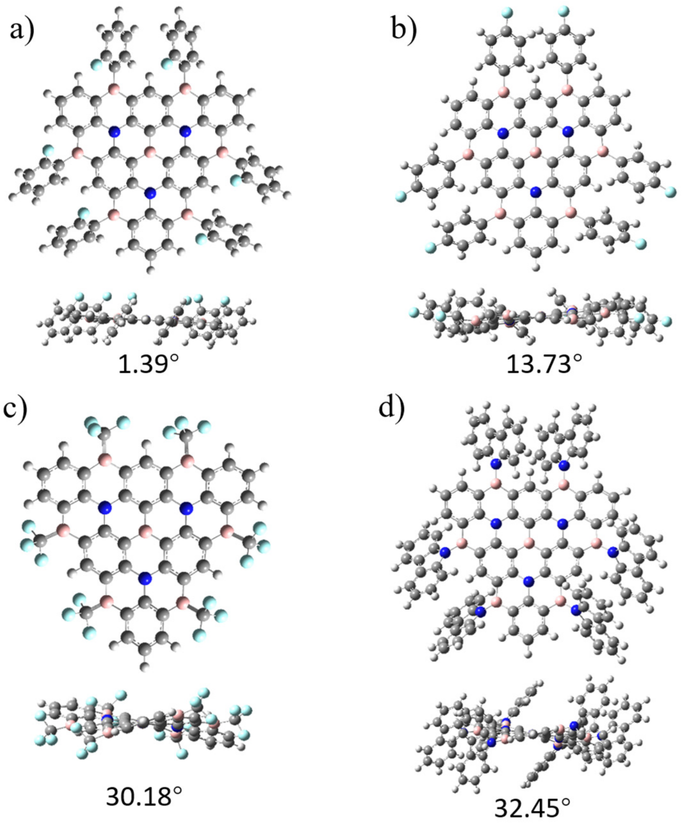

Figure S6. The structure and twist angle of Core3-2F (a), Core3-4F (b), Core3-CF3 (c) and Core3-CZ (d).

4. Triplet State and delta Est

For Vertical Energy of Est

Key words: #p o3lyp/6-311g(d) TD(triplet) IOp(9/40=4)

multiplicity = 1

For Adiabatic Energy of Est

Key words: #p opt freq 6-31g(d) o3lyp

multiplicity = 3

Table S3. Vertical and adiabatic delta Est values of Core3, DABNA-1 and ν-DABNA.

Compound	optimized structure	transition	energy /eV	E	energy Hartree/eV
Core3	S0	S0-S1	2.9917	S0	-1721.360863
		S0-T1	2.6957	S1	-1720.701951
	S1	S1-S0	3.1323	T1	-1720.710675
		Vertical energy	0.293	Adiabatic Energy	0.237277568
DABNA-1	S0	S0-S1	2.9468	S0	-1290.408979
		S0-T1	2.5141	S1	-1289.924902
	S1	S1-S0	2.7014	T1	-1289.936194
		Vertical energy	0.4327	Adiabatic Energy	0.307143216
ν-DABNA	S0	S0-S1	2.7027	S0	-3383.588534
		S0-T1	2.4311	S1	-3382.467873
	S1	S1-S0	2.6471	T1	-3382.476486
		Vertical energy	0.2716	Adiabatic Energy	0.234269792

5. Hole and Electron Analysis

Brief introduction of the hole-electron analysis: ***D***, ***Sr***, ***t***, ***HDI***, ***EDI*** indexes are defined below. [S1]

1. ***D***

The total magnitude of charge transfer (CT) length is referred to as D index:

$$\boldsymbol{D} = \sqrt{\boldsymbol{D_x} + \boldsymbol{D_y} + \boldsymbol{D_z}}$$

where $\boldsymbol{D_X}$, $\boldsymbol{D_Y}$ and $\boldsymbol{D_Z}$ represent the distances between the centroid of hole and electron in corresponding directions.

2. ***Sr***

To characterize overlapping extent of hole and electron, ***Sr*** index are defined as follows:

$$\boldsymbol{S_r} = \int \boldsymbol{S_r}(\boldsymbol{r})d\boldsymbol{r} = \int \sqrt{\boldsymbol{\rho}^{\boldsymbol{hole}}(\boldsymbol{r})\boldsymbol{\rho}^{\boldsymbol{ele}}(\boldsymbol{r})}d\boldsymbol{r}$$

where $\boldsymbol{\rho}^{\boldsymbol{hole}}$ and $\boldsymbol{\rho}^{\boldsymbol{ele}}$ are the hole and electron densities.

3. ***t, H***

t is designed to measure separation degree of hole and electron in CT direction:

$$\boldsymbol{t} = \boldsymbol{D} - \boldsymbol{H_{CT}}$$

$$\boldsymbol{H_{CT}} = |\,(\,|\boldsymbol{\sigma}_{ele}| + |\boldsymbol{\sigma}_{hole}|\,)/2 \cdot \boldsymbol{u}_{CT}\,|$$

where $\boldsymbol{u}_{CT}$ is unit vector in CT direction and can be straightforwardly derived using centroid of hole and electron. The $|\boldsymbol{\sigma}_{ele}|$ and $|\boldsymbol{\sigma}_{hole}|$ are referred to as $\boldsymbol{\sigma}_{ele}$ and $\boldsymbol{\sigma}_{hole}$ indices, they measure overall RMSD of hole and electron, respectively.

4. ***HDI, EDI***

The hole delocalization index (***HDI***) and electron delocalization index (***EDI***) are defined as follows:

$$\boldsymbol{HDI} = 100 \times \sqrt{\int [\boldsymbol{\rho}^{\boldsymbol{hole}}(\boldsymbol{r})]^2 d\boldsymbol{r}}$$

$$\boldsymbol{EDI} = 100 \times \sqrt{\int [\boldsymbol{\rho}^{\boldsymbol{ele}}(\boldsymbol{r})]^2 d\boldsymbol{r}}$$

References

[S1] Lu, T.; Chen, F., Multiwfn: A multifunctional wavefunction analyzer. J Comput Chem 2012, 33, 580-592.
